# Septic Cardiomyopathy in the ICU: Echocardiographic Phenotypes, Global Longitudinal Strain, and Right Ventricular Assessment

**DOI:** 10.3390/diagnostics16111664

**Published:** 2026-05-28

**Authors:** Saeed Torabi, Philipp K. Omuro, Elisabeth H. Adam, Tobias Kammerer

**Affiliations:** Department of Anaesthesiology and Intensive Care Medicine, Faculty of Medicine, University Hospital of Cologne, University of Cologne, 50937 Cologne, Germany; philipp.omuro@uk-koeln.de (P.K.O.); elisabeth.adam@uk-koeln.de (E.H.A.);

**Keywords:** septic cardiomyopathy, echocardiography, global longitudinal strain, right ventricular dysfunction, diastolic dysfunction, speckle-tracking, intensive care unit, hemodynamic monitoring

## Abstract

**Background/Objectives:** Septic cardiomyopathy (SC) is a frequent and clinically relevant complication of sepsis, characterized by acute, typically reversible myocardial dysfunction in the absence of primary coronary artery disease. Its clinical presentation is highly heterogeneous, and conventional diagnostic parameters frequently fail to capture the full extent of cardiac impairment. This narrative review aims to synthesize current evidence on the echocardiographic assessment of SC, with a focused emphasis on global longitudinal strain (GLS), right ventricular (RV) dysfunction, and diastolic impairment, and to discuss their prognostic and therapeutic implications. **Methods:** A comprehensive literature search was performed in PubMed/MEDLINE for publications between 2010 and March 2026, supplemented by seminal historical studies. A second, structured literature search was performed in PubMed/MEDLINE for publications between 2010 and April 2026, supplemented by seminal historical studies. Eligible study types included original research articles, systematic reviews, meta-analyses, and clinical guidelines. The search and selection workflow followed the principles of PRISMA-ScR. A qualitative narrative synthesis was performed. **Results:** SC encompasses distinct echocardiographic phenotypes—global hypokinesia, hyperdynamic function, RV dysfunction, and diastolic impairment—each carrying specific pathophysiological and prognostic implications. Left ventricular ejection fraction (LVEF) is highly load-dependent and may be misleading in vasoplegic states. GLS derived from speckle-tracking echocardiography defects may detect subclinical myocardial dysfunction earlier than LVEF, and provides independent prognostic information in observational studies. RV dysfunction occurs in 20–50% of sepsis patients and represents a powerful robust independent predictor of mortality. Diastolic dysfunction Grade ≥ 2 is independently associated with adverse outcomes through elevated filling pressures and reduced cardiac reserve. **Conclusions:** A multiparametric echocardiographic approach integrating LVEF, GLS, RV function indices, and diastolic parameters is essential and may improve the accurate phenotyping and hemodynamic management of SC. Structured diagnostic algorithms, expert-based diagnostic frameworks incorporating these tools which may facilitate phenotype-guided therapy and improve clinical outcomes, are proposed to facilitate phenotype-guided therapy; their impact on patient outcomes warrants confirmation in prospective multicenter studies.

## 1. Introduction

Sepsis is defined by the Sepsis-3 consensus criteria as life-threatening organ dysfunction arising from a dysregulated host response to infection, operationalized by an acute increase in the Sequential Organ Failure Assessment (SOFA) score of ≥2 points [1,2]. Globally, sepsis accounts for approximately 11 million deaths annually and affects nearly two million patients per year in the United States alone, with an overall in-hospital mortality of approximately 25%, rising to 40–70% for septic shock [2,3].

A central and pathophysiologically complex consequence is septic cardiomyopathy, also termed sepsis-induced myocardial dysfunction, first described in the 1980s by Parker et al. as profound but reversible myocardial depression in patients with septic shock [4]. SC currently affects an estimated 40–60% of critically ill sepsis patients and is characterized by acute, reversible biventricular dysfunction in the absence of acute coronary syndrome (ACS) or pre-existing structural heart disease [5,6].

Despite its high clinical prevalence, SC lacks a universally accepted definition. Heterogeneity in diagnostic criteria—particularly EF thresholds and echocardiographic timing—has substantially complicated research and clinical recognition [7,8]. Clinical presentation spans a broad spectrum: from subclinical echocardiographic abnormalities to overt biventricular failure and paradoxically hyperdynamic states, each with distinct pathophysiological mechanisms and prognostic trajectories.

Echocardiography has emerged as the primary bedside modality for cardiac assessment in septic patients, enabling a real-time, non-invasive evaluation of ventricular function, filling pressures, and hemodynamic status [9]. While LVEF remains widely used, its load dependency fundamentally limits its utility as a contractility index in sepsis. Advanced imaging modalities, most notably GLS derived from speckle-tracking echocardiography (STE), may offer superior sensitivity for subclinical myocardial dysfunction and provide prognostic information independent of LVEF [10,11].

RV dysfunction, occurring in up to 50% of sepsis patients, is driven by pulmonary vascular disease, ARDS-related afterload increase, and direct myocardial depression, and represents the strongest independent echocardiographic predictor of mortality reported in this population [12,13]. Diastolic dysfunction, frequently present irrespective of systolic function, contributes to adverse outcomes through elevated filling pressures and impaired cardiac reserve [14].

This narrative review synthesizes current evidence on the pathophysiology and echocardiographic characterization of SC, with a focused emphasis on GLS, RV function assessment, and diastolic dysfunction, and discusses the integration of these findings into structured diagnostic frameworks and hemodynamic management strategies.

## 2. Methods

This narrative review followed the principles of structured narrative synthesis with reporting in line with the PRISMA-ScR (Preferred Reporting Items for Systematic Reviews and Meta-Analyses extension for Scoping Reviews) framework as a transparency guide. The completed PRISMA-ScR flow diagram and checklist are provided as Supplementary File S1; a structured summary of the principal included clinical studies is provided as Supplementary Table S1.

Information sources and search strategy. PubMed/MEDLINE was searched as the principal information source. The final literature search was last updated on 15 April 2026. Search terms (Boolean-combined where appropriate) included: “septic cardiomyopathy”, “sepsis-induced myocardial dysfunction”, “global longitudinal strain sepsis”, “speckle-tracking echocardiography sepsis”, “right ventricular dysfunction sepsis”, “diastolic dysfunction sepsis”, “hemodynamic monitoring sepsis”, and “septic shock echocardiography”. Searches were restricted to English language publications. The principal time frame was 2010 to April 2026; seminal earlier publications (e.g., [4,15]) were retained where they provided foundational mechanistic or historical context.

Eligibility criteria. Eligible study types included original research articles (prospective and retrospective cohort studies), randomized controlled trials, systematic reviews and meta-analyses, clinical practice guidelines and consensus statements, and translational studies of high clinical relevance to the ICU management of SC. Exclusion criteria included case reports and small case series without broadly generalizable findings, pediatric-only cohorts (where adult-relevant data were unavailable), purely animal studies without translational relevance, conference abstracts without peer-reviewed full-text publication, and non-English publications.

Selection process. Two authors (S.T. and P.K.O.) independently screened the titles and abstracts of all retrieved records. Potentially eligible articles were retrieved in full text and assessed against the eligibility criteria above. Disagreements were resolved by consensus discussion involving a third author (T.K.). When multiple publications addressed the same evidence base, the most recent and methodologically rigorous source was preferred.

Prioritization hierarchy. Within the eligible studies, the following prioritization hierarchy was applied for narrative synthesis: (i) high-quality systematic reviews and meta-analyses; (ii) randomized controlled trials and large multicenter prospective cohort studies; (iii) smaller prospective cohort studies and translational studies; and (iv) mechanistic and pathophysiological studies of high clinical relevance. International guideline documents (Surviving Sepsis Campaign, ASE/EACVI consensus statements) were always included.

Selection process outcome. Approximately 1250 records were initially identified across the structured search strings. After removal of duplicates and title/abstract screening, approximately 410 records were retained for full-text review. Of these, approximately 180 full-text articles were assessed for eligibility, and 69 publications were ultimately included in the qualitative synthesis (61 references retained from the originally submitted manuscript and 8 references added in revision to address reviewer comments). The flow of records through the selection process is summarized in Supplementary File S1.

Synthesis. Data were synthesized qualitatively and structured by clinical theme, as follows: definitions, epidemiology, pathophysiology, echocardiographic phenotypes, diagnostic strategies, biomarkers, and therapeutic considerations. Given the substantial heterogeneity of study populations, diagnostic criteria, and outcome definitions across the eligible literature, no formal quantitative meta-analytic synthesis was performed. The strength of recommendations made throughout this review is calibrated to the underlying evidence: where applicable, recommendations are explicitly identified as evidence-supported, physiology-based/expert opinion, or supported by negative or insufficient interventional evidence (Section 9; Supplementary Table S2).

## 3. Sepsis Definitions and Cardiac Phenotypes

### 3.1. Sepsis-3 Classification Framework

The 2016 Sepsis-3 consensus replaced the prior SIRS-based definition with an organ dysfunction-centered framework. Sepsis is defined by a SOFA score increase of ≥2 points in the context of suspected infection. Septic shock is defined by the requirement for vasopressors to maintain MAP ≥ 65 mmHg following adequate volume resuscitation combined with a serum lactate >2 mmol/L, conferring a hospital mortality exceeding 40% [1,2].

### 3.2. Echocardiographic Phenotypes of Septic Cardiomyopathy

Cardiac dysfunction in sepsis encompasses multiple phenotypically and prognostically distinct manifestations [5,7]. The phenotypes are shown in Table 1. The following five principal echocardiographic phenotypes are recognized: (1) global biventricular hypokinesia (classic SC presentation, LVEF typically <45–50%, reversible within 7–10 days); (2) hyperdynamic function (supranormal LVEF > 60–75% in vasoplegic distributive shock—not reflecting preserved contractility); (3) Takotsubo-like pattern (apical ballooning with basal hyperkinesia, catecholamine-mediated); (4) diastolic dysfunction (frequently independent of systolic LVEF; Grade ≥ 2 carries independent prognostic significance); and (5) RV dysfunction (present in 20–50% of cases, a strong independent mortality predictor in observational studies).

## 4. Epidemiology and Prognosis

### 4.1. Incidence

The reported prevalence of SC varies widely, from 14% to 60% across published series [6,16,17,18]. This heterogeneity reflects the absence of a consensus definition, divergence in EF thresholds, and the timing of echocardiographic assessments. Studies performed within the first 6 h of sepsis onset systematically underestimate incidence, as myocardial dysfunction typically manifests fully between 6 h and 72 h after sepsis onset [6,17,18].

### 4.2. Phenotype-Specific Prognostic Profiles

The prognostic implications of cardiac dysfunction in sepsis are phenotype-dependent (Table 2). Isolated LV systolic dysfunction with reduced LVEF does not confer significantly elevated short-term mortality. The compensatory LV dilatation characteristic of classic SC maintains stroke volume through the Frank–Starling mechanism, while simultaneously reducing wall tension and myocardial oxygen demand [19,20,21]. Hyperdynamic physiology paradoxically confers elevated mortality, reflecting the severity of underlying vasoplegic shock [19,22,23].

RV dysfunction is the most robust echocardiographic predictor of adverse outcomes in observational studies. A prospective cohort study demonstrated a 3.4-fold increase in 28-day mortality in patients with RV dysfunction, while LV systolic dysfunction showed no significant mortality association in the same cohort [12]. A meta-analysis of 10 studies (*n* = 1373) confirmed a 2.4-fold elevation in short-term and a 2.3-fold elevation in long-term mortality attributable to RV dysfunction [13]. Diastolic dysfunction Grade ≥ 2 is an independent predictor of mortality mediated by elevated filling pressures [14,15]. SC demonstrates marked phenotypic instability: in one cohort, the phenotype changed between admission and discharge in 89% of initially hypokinetic and 86% of initially hyperdynamic patients [24].

## 5. Pathophysiology

SC is driven by a complex, multifactorial interplay of inflammatory, mitochondrial, and microvascular mechanisms rather than primary ischemia—global coronary perfusion is characteristically increased in sepsis (see Table 3) [6,25].

### 5.1. Cytokine-Mediated Myocardial Depression

Pathogen-associated molecular patterns (PAMPs) and damage-associated molecular patterns (DAMPs) activate Toll-like receptors (TLRs) on cardiomyocytes, triggering release of TNF-α, IL-1β, and IL-6. These upregulate inducible nitric oxide synthase (iNOS), causing excessive nitric oxide (NO) production. At high concentrations, NO inhibits mitochondrial respiratory chain complexes I–IV and exerts negative inotropic effects. Interaction with superoxide anions generates peroxynitrite, irreversibly nitrosylating contractile proteins [26,27,28].

### 5.2. Mitochondrial Dysfunction and Energetic Failure

Reactive oxygen species (ROS) selectively inhibit mitochondrial electron transport chain complexes, causing a dramatic reduction in ATP synthesis. The resulting energetic failure impairs actomyosin cross-bridge cycling and calcium-dependent excitation-contraction coupling. This mitochondrial dysfunction is fully reversible in survivors—mediated by mitochondrial biogenesis and selective autophagy (mitophagy)—fundamentally distinguishing SC from ischemic cardiomyopathy [29,30].

### 5.3. Calcium Homeostasis and Beta-Adrenergic Desensitization

Septic mediators reduce sarcolemmal L-type Ca^2+^ channel density and impair SERCA2a activity, disrupting diastolic calcium reuptake and reducing troponin–calcium sensitivity, resulting in diminished systolic contractility and prolonged diastolic relaxation [16,26,31]. Sustained catecholamine excess downregulates myocardial β1- and β2-adrenoceptors through receptor internalization and adenylate cyclase uncoupling, progressively blunting the inotropic response [32,33].

### 5.4. Microvascular Dysfunction and Frank–Starling Adaptation

Despite increased global coronary flow, microvascular dysfunction—characterized by endothelial injury, microthrombosis, and impaired vasoreactivity—generates heterogeneous myocardial perfusion and subendocardial ischemia without macrovascular obstruction [6,25]. Compensatory LV dilatation in classic SC maintains stroke volume through preload augmentation while simultaneously reducing wall tension (Laplace’s law), limiting myocardial oxygen demand—a cardioprotective adaptation explaining the neutral short-term mortality of classical hypokinetic SC [19,20,21].

## 6. Echocardiographic Assessment

### 6.1. Limitations of LVEF in Sepsis

LVEF remains the most widely used marker of LV systolic function, yet its validity as an index of intrinsic contractility is fundamentally limited in sepsis by load dependency. In hyperdynamic states, LVEF may be supranormal (>70–75%) despite significant underlying myocardial dysfunction—a phenomenon of pseudonormalization. Conversely, compensatory LV dilatation may transiently maintain LVEF through preload augmentation without reflecting improved contractility [20,22,34]. LVEF should therefore be interpreted in the context of loading conditions, ventricular geometry, and complementary parameters of myocardial deformation; the standard chamber quantification methodology has been outlined by the ASE/EACVI consensus [35]. An IVC-based fluid responsiveness assessment is substantially confounded by tidal volume, respiratory mode, intra-abdominal pressure, and cardiac rhythm in mechanically ventilated patients, and must be interpreted in the broader hemodynamic context [36,37].

### 6.2. Global Longitudinal Strain: Principles and Clinical Application

Speckle-tracking echocardiography provides an angle-independent quantitative myocardial deformation analysis by tracking the frame-by-frame displacement of acoustic speckles within the myocardium [38]. GLS quantifies the percentage shortening of the LV myocardium in the longitudinal axis from end-diastole to end-systole, averaged across all 17 segments. Values are expressed as negative percentages; fewer negative values indicate impaired function [38,39,40]. Reference ranges are −18% to −22% in healthy adults [40,41]. Inter-vendor algorithmic variability is well documented, with absolute differences of up to 3–4 percentage points between platforms; the reader is referred to the EACVI/ASE/Industry Task Force consensus document on STE standardization and to the EACVI/ASE Inter-Vendor Comparison Study for technical details [42,43].

In observational studies, GLS consistently outperforms LVEF in sensitivity for early myocardial dysfunction detection in sepsis. In the SPECKSS prospective case control study, patients with septic shock demonstrated significantly impaired GLS despite comparable LVEF to controls [44]. GLS abnormalities precede LVEF decline by 12–24 h in serial assessments, potentially enabling earlier therapeutic intervention [45]. A multicenter cohort analysis confirmed that abnormal GLS at ICU admission independently predicted long-term mortality, ICU length of stay, and vasopressor requirements beyond LVEF [46,47].

The “hidden strain loss” phenomenon in hyperdynamic SC is particularly instructive: patients with supranormal LVEF frequently demonstrate significantly impaired GLS, reflecting disrupted longitudinal contractility despite compensatory preservation of radial and circumferential function. GLS thus reveals myocardial impairment masked by the apparent normality of LVEF in vasoplegic states [34]. Based on the available—predominantly observational—evidence, GLS should be considered as a complementary parameter alongside LVEF where technically feasible and where local expertise and software platforms support standardized acquisition [11]. The practical limitations of GLS acquisition in critically ill patients, including image-quality constraints, tachycardia, arrhythmia, and inter-vendor variability, are addressed in Section 6.5. A summary of GLS values can be seen in Table 4.

### 6.3. Right Ventricular Assessment

#### 6.3.1. Anatomical Vulnerability and Pathophysiological Context

The RV differs fundamentally from the LV in anatomy and functional physiology. Its thin wall (2–5 mm), complex crescentic geometry, and optimization for high-volume, low-pressure loading render it acutely vulnerable to afterload increases: a doubling of pulmonary artery pressure can rapidly precipitate RV decompensation [12]. In sepsis, the RV faces a convergence of adverse loading conditions: hypoxia-induced pulmonary vasoconstriction, inflammation-mediated pulmonary vascular resistance elevation, ARDS-related parenchymal injury, high ventilatory driving pressures, and direct septic myocardial depression [6,12].

#### 6.3.2. Prevalence and Prognostic Significance

RV dysfunction occurs in 20–50% of sepsis patients. The University of Utah cohort documented a 3.4-fold increase in 28-day mortality with RV dysfunction, while LV systolic dysfunction carried no significant mortality signal [12]. The meta-analysis by Vallabhajosyula et al. (10 studies, *n* = 1373) confirmed a 2.4-fold elevated short-term and a 2.3-fold elevated long-term mortality [13]. Mechanistically, RV failure reduces LV preload through ventricular interdependence, induces leftward interventricular septal shift (D-sign), restricts LV filling through pericardial constraint, and elevates myocardial oxygen demand at reduced coronary perfusion pressure [6,12,48].

#### 6.3.3. Echocardiographic Multiparametric Protocol

RV function should be assessed using a structured multiparametric approach whenever feasible. TAPSE (≥17 mm; M-mode) reflects longitudinal RV systolic excursion but is subject to preload dependency (Table 5) [49,50]. TDI S’ velocity (≥9.5 cm/s at the lateral tricuspid annulus) is less preload-dependent but angle-limited [49]. FAC (≥35%; apical four-chamber planimetry) captures both longitudinal and radial contractile components and is less preload-sensitive [51]. The RV/LV ratio (normal < 2/3) identifies RV dilatation; a focused RV-centered four-chamber view is preferred to avoid transducer positioning artifacts. The D-sign on the parasternal short-axis identifies leftward septal shift: systolic flattening indicates pressure overload; diastolic flattening indicates volume overload [52]. RV Free Wall Strain (RVFWS; normal −20% to −28%) by STE is a sensitive parameter for early RV dysfunction and correlates with mortality in sepsis cohorts even when TAPSE and FAC remain borderline [53]; however, RVFWS feasibility in the ICU is limited (Section 6.5). The standardized acquisition methodology for RV chamber quantification is detailed in the ASE/EACVI consensus [35].

#### 6.3.4. Differential Diagnosis and RV-Targeted Management

The differential diagnosis of acute RV dysfunction in sepsis includes acute pulmonary embolism (PE), ARDS-associated right heart strain, COPD exacerbation, RV myocardial infarction, and ventilator-induced RV afterload increase. The 60-60 rule provides 94% specificity for PE; RV wall thickness (>5 mm indicates chronic hypertrophy) helps differentiate acute from chronic RV overload. RV-targeted management strategies include minimization of driving pressure and PEEP within an RV-protective ventilation framework [54,55], restrictive fluid strategy with consideration of decongestive therapy, norepinephrine to maintain systemic MAP exceeding mean pulmonary artery pressure, dobutamine or milrinone for impaired RV contractility, inhaled NO or prostacyclin for elevated pulmonary vascular resistance, and veno-arterial ECMO as a bridge-to-recovery in refractory RV failure [56,57]. These RV-targeted recommendations are predominantly physiology-based and supported by observational data from ARDS cohorts; phenotype-specific randomized evidence in sepsis remains limited (Section 9; Supplementary Table S2).

### 6.4. Diastolic Dysfunction

Diastolic dysfunction in sepsis arises predominantly from impaired SERCA2a-mediated calcium reuptake and increased LV stiffness, causing elevated end-diastolic filling pressures and impaired ventricular filling—frequently independent of systolic LVEF [6,14]. An echocardiographic diastolic assessment incorporates the transmitral E/A ratio, the septal and lateral early diastolic annular velocity (e’) by TDI, the E/e’ ratio as a surrogate for LV filling pressure, and the left atrial volume index (LAVI). An E/e’ >14 indicates elevated LV filling pressures (PCWP > 15 mmHg); a lateral e’ < 9 cm/s denotes significant diastolic impairment [14,58]. The full ASE/EACVI diastolic grading algorithm was updated in 2016 [59].

Application of these grading criteria in the ICU is limited by tachycardia-related E–A fusion and ventilator-induced stroke volume variation [14]. Despite these limitations, diastolic dysfunction Grade ≥ 2 is an independent mortality predictor across multiple meta-analyses [14,15]. Elevated E/e’ following volume resuscitation serves as a clinically valuable predictor of fluid non-response and pulmonary edema risk, and may be integrated into hemodynamic decision-making [58].

### 6.5. Practical Limitations and Feasibility in the ICU

Advanced echocardiographic techniques—GLS, RVFWS, and quantitative diastolic grading—offer substantial diagnostic and prognostic information in sepsis, but their applicability in the critical care environment is limited by several technical, physiological, and operator-related factors that should be considered when interpreting findings.

Image quality constraints. Acoustic windows in critically ill patients are frequently suboptimal due to mechanical ventilation (positive intrathoracic pressure, lung hyperinflation), subcutaneous emphysema, surgical dressings or drains, prone or semi-recumbent positioning, and obesity. Reported feasibility rates for standard GLS acquisition in mixed ICU cohorts range from approximately 70% to 85%; for RVFWS, feasibility is lower (60–80%) due to the complex RV geometry, foreshortened views, and acoustic dropout in the RV apex.

Heart rate, rhythm, and load. Tachycardia at heart rates >90–100 bpm produces E–A fusion that disproportionately compromises conventional diastolic grading, while arrhythmias—particularly atrial fibrillation—introduce beat-to-beat variability that affects the reliability of strain and Doppler-based measurements; averaging over multiple cardiac cycles partially mitigates but does not eliminate this limitation. All conventional and strain-based parameters are load-dependent to varying degrees; rapid changes in volume status, vasopressor titration, and ventilator settings during ongoing resuscitation can produce apparent improvement or deterioration that does not reflect intrinsic myocardial function.

Inter-vendor and inter-observer variability. GLS measurements vary by 3–4 percentage points between different speckle-tracking platforms, and absolute thresholds derived on one platform should be applied with caution to another [42,43]. Inter-observer variability for advanced parameters typically range from 5% to 10% in trained operators, and is higher in less experienced examiners; standardized acquisition protocols, dedicated training, and routine quality assurance reduce but do not eliminate this variability.

Implications for clinical use. Given these limitations, advanced echocardiographic parameters should be interpreted as complementary rather than stand-alone clinical findings, in conjunction with the broader hemodynamic, ventilatory, and clinical context. Where technically feasible, a longitudinal serial assessment within an individual patient and on a single platform is more informative than a cross-sectional comparison against external thresholds. Comprehensive operator training and standardized protocols for image acquisition and analysis are prerequisites for reliable use in the ICU.

## 7. Diagnostic Protocol and Echocardiographic Algorithm

### 7.1. Indications and Timing

Routine echocardiographic monitoring of all sepsis patients is not supported by current evidence. Echocardiography is indicated in persistent hemodynamic instability despite adequate volume and vasopressor therapy, in diagnostic uncertainty between distributive and cardiogenic shock, in new cardiac findings (murmur, arrhythmia, unexplained troponin elevation), and in serial follow-up to document myocardial recovery. Assessments within the first 6 h underestimate the SC prevalence; serial echocardiography at 24–72 h provides an essential phenotypic re-evaluation for all patients with confirmed SC [22].

### 7.2. Structured Echocardiographic Protocol

A complete sepsis echocardiographic protocol incorporates the following: LVEF by the biplane Simpson method; GLS by STE in three apical planes (where feasible); LVOT-VTI and stroke volume calculation; diastolic parameters (E/A, e’, E/e’, LAVI); TAPSE, S’, FAC, RV/LV ratio, D-sign, and RVFWS where feasible; IVC diameter and collapsibility index with contextual interpretation under ventilation; and estimated PASP from peak TR velocity. The standardized acquisition methodology for chamber quantification follows the ASE/EACVI consensus [35], and the diastolic assessment follows the 2016 ASE/EACVI recommendations [59].

### 7.3. Step-Wise Diagnostic Algorithm

**Figure 1:**
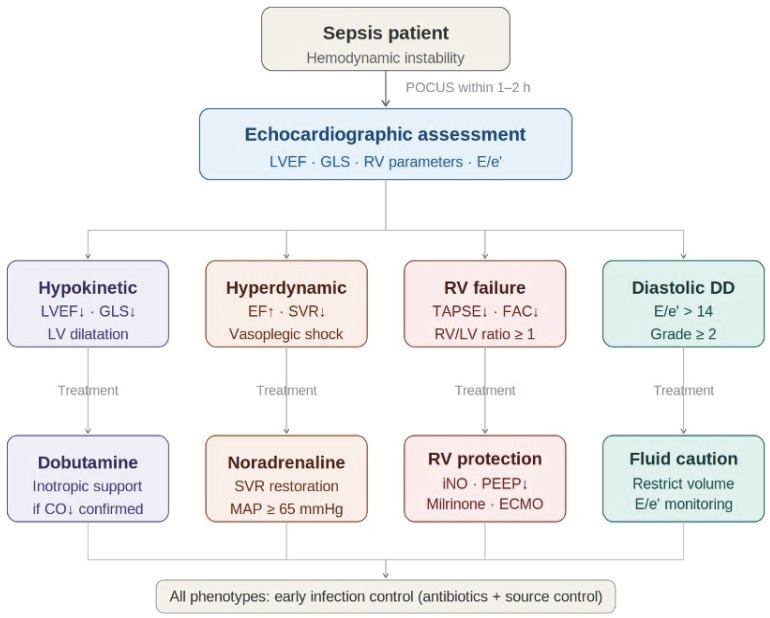
Proposed conceptual framework for phenotype-guided echocardiographic assessment and management for septic cardiomyopathy in the ICU. This framework integrates physiological reasoning and observational evidence, and is intended as a clinical decision support tool; it has not been prospectively validated against patient-centered outcomes. All phenotypes require early infection control as the causal therapeutic priority. Evidence grading for each therapeutic branch is provided in Supplementary Table S2. CO = cardiac output; ECMO = extracorporeal membrane oxygenation; iNO = inhaled nitric oxide; MAP = mean arterial pressure; PEEP = positive end-expiratory pressure; SVR = systemic vascular resistance.

The following pragmatic, expert consensus-based framework is proposed for hemodynamically unstable sepsis patients not responding to standard resuscitation (Figure 1). It has not been prospectively validated as a clinical pathway, and its impact on patient outcomes warrants future evaluation in randomized trials. Evidence grading for the therapeutic branches of Figure 1 is provided in Supplementary Table S2.

(1) Clinical contextualization: cardiac history, known baseline LVEF, current perfusion parameters (lactate, ScvO_2_, capillary refill time, urine output, vasopressor requirements).
(2) POCUS within 1–2 h: global LV/RV function, LVEF estimation, IVC, pericardial effusion/tamponade, pneumothorax exclusion.
(3) Complete sepsis echocardiography (Table 6) including RV multiparametric assessment, diastolic evaluation, and GLS where feasible.
(4) Biomarkers: high-sensitivity troponin, NT-proBNP, serum lactate, ScvO_2_.
(5) 12-lead ECG: STEMI exclusion, ischemic pattern, arrhythmia.
(6) Advanced hemodynamic monitoring (transpulmonary thermodilution or pulmonary artery catheter) in persistent instability or complex biventricular/mixed shock.
(7) Coronary angiography only when clinical ACS is suspected (STEMI pattern, regional wall motion abnormality, hemodynamically stable patient).
(8) Serial echocardiography at 24–72 h for phenotype re-evaluation and recovery documentation.

## 8. Biomarkers in Septic Cardiomyopathy

Cardiac biomarkers provide complementary diagnostic and prognostic information in SC, though their specificity is substantially limited by the systemic inflammatory milieu of sepsis. High-sensitivity troponin I/T is elevated in 85–95% of all sepsis patients irrespective of echocardiographically confirmed SC, reflecting generalized cardiomyocyte stress, demand ischemia, and cytokine-mediated injury [60]. Despite limited specificity, peak troponin values carry significant prognostic associations with 1-year cardiovascular events [60].

NT-proBNP and BNP reflect myocardial wall stress and are useful for serial trajectory monitoring. In a meta-analysis of 36 studies (approximately 3500 patients), BNP > 620 pg/mL and NT-proBNP > 4000 pg/mL were associated with 28-day mortality (sensitivity ~70%, specificity ~91%); early measurements within 24 h provided superior prognostic discrimination [6,61]. Confounders include renal insufficiency, RV pressure overload in ARDS, and aggressive volume resuscitation. Serial lactate monitoring reflects global tissue hypoperfusion and remains a powerful independent mortality predictor [1,2].

## 9. Therapeutic Considerations

Therapeutic recommendations for SC are presented below in three explicit evidence categories, as follows: (i) evidence-supported recommendations (guideline-grade evidence or robust randomized controlled trials); (ii) physiology-based/expert opinion recommendations (derived from pathophysiological reasoning and observational evidence, but not from phenotype-stratified outcome trials); and (iii) interventions with negative or insufficient trial evidence. The same categorization is applied to each therapeutic branch of Figure 1 in Supplementary Table S2.

### 9.1. Causal Therapy: Infection Control (Evidence-Supported)

The cornerstone of SC management is prompt control of the underlying infection. The 2021 Surviving Sepsis Campaign guidelines advocate initiation of broad-spectrum antimicrobial therapy within one hour of diagnosis alongside early source control [62]. Myocardial recovery closely parallels infection resolution; no cardiac-specific intervention supersedes causal therapy in clinical priority.

### 9.2. Evidence-Supported Hemodynamic Management

Norepinephrine is the vasopressor of first choice in septic shock, targeting MAP ≥ 65 mmHg, supported by the Surviving Sepsis Campaign guidelines as a strong recommendation [62]. In hyperdynamic SC, norepinephrine is particularly critical for SVR restoration. Vasopressin may be added adjunctively for norepinephrine-sparing benefit [62]. Volume resuscitation should be individualized using dynamic fluid-responsiveness tests rather than static measures: passive leg raising, end-expiratory occlusion, and stroke volume responses to a fluid challenge have robust meta-analytic evidence of superior diagnostic performance over CVP and IVC alone [36,63]. Hydrocortisone is recommended in vasopressor-dependent septic shock; it reduces vasopressor duration and requirements, while a direct myocardioprotective effect has not been established [64,65].

### 9.3. Physiology-Based and Expert Opinion Recommendations

Several clinically important interventions in SC are predominantly physiology-based, supported by observational data or by expert consensus, but have not been demonstrated to reduce mortality in dedicated phenotype-stratified randomized controlled trials in sepsis.

Inotropic support in hypokinetic SC. Dobutamine is recommended by the Surviving Sepsis Campaign on a conditional basis when persistent hypoperfusion exists despite adequate fluid status and MAP [62]. Risks include tachycardia, increased myocardial oxygen demand, and arrhythmia; dobutamine is contraindicated in hyperdynamic SC, where supranormal LVEF reflects vasoplegia rather than preserved cardiac reserve [17,18].

RV-targeted strategies. RV-protective ventilation (minimization of driving pressure and PEEP; permissive hypercapnia where clinically appropriate) is supported by extensive observational and physiological evidence in ARDS [54,55]. Inhaled nitric oxide or prostacyclin acutely reduces pulmonary vascular resistance and may improve RV performance, but no dedicated sepsis RCT has demonstrated outcome benefit [54,56]. The restrictive volume strategy in established RV dilatation is a physiologically grounded recommendation supported by observational data. Veno-arterial ECMO may be considered as a bridge-to-recovery in refractory cardiogenic SC when the infection source has been controlled and myocardial recovery is anticipated; however, the evidence base for sepsis-related cardiogenic shock remains limited [57].

Volume management in diastolic dysfunction. In phenotypes with elevated filling pressures (E/e’ > 14, Grade ≥ 2 diastolic dysfunction), a restrictive volume strategy with serial E/e’ monitoring after fluid challenges is advocated to limit pulmonary edema risk and the worsening of RV function. This is supported by observational data; no randomized phenotype-stratified trial of E/e’-guided fluid strategy has been performed in sepsis [14,58].

### 9.4. Interventions Without Established Mortality Benefit

Beta-blockers (esmolol, landiolol). Initial single-center RCT were not confirmed in subsequent multicenter trials, including the STRESS-L trial. A systematic review with trial sequential analysis found no robust evidence of mortality benefit from either esmolol or landiolol, and identified a signal of hemodynamic deterioration [66]. Beta-blockers are therefore not currently recommended outside investigational settings.

Levosimendan. The LeoPARDS randomized trial and its subsequent subgroup analyses showed no mortality benefit in septic shock; levosimendan is therefore not currently recommended as standard therapy for SC [67].

## 10. Discussion

### 10.1. Interpretation of Key Findings

The present review highlights several clinically important insights regarding echocardiographic phenotyping in septic cardiomyopathy. The paradoxical dissociation between LVEF and outcomes is one of the most clinically consequential findings in this field: hyperdynamic function, despite supranormal LVEF, carries elevated mortality—a counterintuitive relationship that reflects the severity of underlying vasoplegic physiology rather than preserved myocardial integrity. This has direct implications for bedside interpretation: clinicians must resist the reassuring appearance of a vigorously contracting ventricle and recognize it as a potential marker of severe distributive shock requiring vasopressor optimization rather than inotropic support.

Second, GLS has emerged as a sensitive and prognostically informative parameter that may outperform LVEF in detecting early myocardial dysfunction—particularly in the hyperdynamic state where conventional LVEF appears falsely normal. The 12–24 h observational advantage of GLS over LVEF for detecting incipient deterioration may offer a window for earlier therapeutic intervention. Third, RV dysfunction deserves recognition as one of the strongest echocardiographic predictors of adverse outcomes in sepsis observational studies, yet it remains underappreciated and underassessed in routine clinical practice—often evaluated with TAPSE alone rather than the comprehensive multiparametric protocol warranted by its prognostic importance.

### 10.2. Clinical Implications

The clinical implications of these findings are substantial. For intensivists, the systematic integration of GLS into echocardiographic protocols where technically feasible may enable earlier identification of patients at risk of clinical deterioration and inform earlier hemodynamic interventions. Adopting a multiparametric RV assessment—where feasible, rather than relying on TAPSE alone—may substantially improve risk stratification, since each RV parameter captures a distinct dimension of RV performance. A diastolic assessment, particularly E/e’ monitoring after volume resuscitation, provides valuable guidance for fluid management decisions and helps limit the recognized harms of excessive volume loading in the vulnerable RV.

The structured framework presented in this review (Figure 1, Section 7.3) offers a practical conceptual approach for integrating these insights into bedside management. Its implementation requires no additional technology beyond standard echocardiographic equipment, making it applicable in any ICU with echocardiography capability. The ability to stratify patients into distinct phenotypic categories enables the rational selection of vasoactive agents—norepinephrine for vasoplegia, dobutamine for true hypokinesia, RV-protective strategies for right heart failure—and limits the one-size-fits-all approach that characterizes much of current practice. We emphasize that this framework is conceptual and expert-based, not prospectively validated; its outcome impact remains to be demonstrated in interventional trials.

### 10.3. Limitations

Several limitations of this review warrant acknowledgment. First, as a narrative review, it is not subject to a formal systematic search protocol or quality assessment of included studies, introducing potential selection bias in the literature synthesized; the PRISMA-ScR-aligned methodology (Section 2; Supplementary File S1) and the supplementary table of key included studies (Supplementary Table S1) are intended to mitigate but not eliminate this risk. Second, no meta-analytic synthesis was performed, given the substantial heterogeneity of study populations, diagnostic criteria, and outcome definitions across the literature. Third, the lack of a universally accepted definition of SC—particularly regarding EF thresholds and measurement timing—limits direct comparability across the studies, and likely contributes to the wide variation in reported prevalence (14–60%). Fourth, GLS values are subject to vendor-specific algorithmic variability between speckle-tracking echocardiography platforms; absolute thresholds discussed in this review should be applied with caution when derived from different vendor systems [42,43], and longitudinal comparisons within an individual patient should ideally use a single platform. Fifth, the evidence base for specific phenotype-guided therapeutic interventions remains limited; most clinical trials in sepsis have not stratified patients by echocardiographic phenotype, making it difficult to draw definitive conclusions about which interventions benefit which phenotypic subgroup. Finally, the practical limitations of advanced echocardiography in the ICU (Section 6.5) constrain the universal applicability of strain-based and three-dimensional approaches.

### 10.4. Future Directions

Future research should focus on several priority areas. The development of internationally standardized definitions for SC—including consensus on EF thresholds, measurement timing, and minimum required echocardiographic parameters—is a prerequisite for meaningful comparative research and clinical trial design. Large, multicenter prospective studies are needed to establish whether the systematic application of GLS and RV free wall strain assessments during sepsis translate into measurable improvements in patient outcomes. The validation of phenotype-specific therapeutic algorithms, integrating echocardiographic phenotyping into the design of randomized controlled trials, represents the most important unmet need in this field; the framework presented in Figure 1 is well-suited as a starting point for such validation.

Three-dimensional echocardiography for RV assessment. The complex crescentic geometry of the RV is poorly captured by two-dimensional surrogates, such as TAPSE and FAC. Three-dimensional echocardiography enables direct volumetric quantification of RV end-diastolic volume, end-systolic volume, RV stroke volume, and true RVEF. Muraru et al. validated a dedicated 3D speckle-tracking algorithm against cardiac magnetic resonance and demonstrated good correlation and acceptable limits of agreement for RV volumes and ejection fraction [68]. The principal advantages of 3D RV echocardiography over conventional 2D parameters include the capture of the full RV geometry, lower load dependency relative to TAPSE, and the ability to track quantitative recovery over time. Limitations relevant to the ICU setting include image-quality dependency, restricted feasibility under mechanical ventilation, the need for dedicated software and trained operators, and longer analysis times. While 3D RV echocardiography is not yet routine in ICU practice, it represents a promising tool for serial RV assessments in dedicated centers and a logical reference standard for future validation studies.

Artificial intelligence-assisted automated GLS analysis. Manual or semi-automatic GLS analysis is time-consuming, requires specialized expertise, and is subject to operator-dependent variability, all of which limit its uptake in routine ICU practice. Salte et al. developed a fully automated deep learning-based GLS pipeline and demonstrated good correlation (R = 0.93) and low bias relative to conventional speckle-tracking, with markedly reduced analysis time and improved test–retest reproducibility [69]. Subsequent real-world clinical service evaluations have confirmed high feasibility (>95%) and substantially reduced inter-measurement variability in patient cohorts. Such platforms have the potential to enable real-time bedside GLS assessment without dedicated strain expertise and to standardize measurements across vendors and centers. Sepsis-specific validation studies remain limited, and the clinical impact of AI-assisted strain on outcomes has not yet been demonstrated; integration of these tools into prospective multicenter sepsis trials is an attractive direction.

## 11. Conclusions

Septic cardiomyopathy is a multifaceted and clinically impactful complication of sepsis, encompassing phenotypically distinct forms of biventricular dysfunction with divergent pathophysiological underpinnings and prognostic trajectories. A defining characteristic is its paradoxical outcome profile: while classic hypokinetic LV dysfunction is frequently associated with neutral short-term mortality through compensatory Frank–Starling adaptation, hyperdynamic physiology—despite a supranormal LVEF—confers elevated mortality as a marker of profound vasoplegic shock rather than preserved myocardial function.

Echocardiography is the central diagnostic modality for bedside phenotyping in sepsis. An LVEF assessment, while necessary, is insufficient in isolation. GLS by speckle-tracking echocardiography may provide superior sensitivity for subclinical myocardial dysfunction, can identify longitudinal impairment masked by pseudonormal LVEF in hyperdynamic states, and offers independent prognostic information in observational studies. GLS should be considered as a complementary parameter alongside LVEF where technically feasible and where local expertise supports standardized acquisition.

RV dysfunction, present in up to 50% of cases, represents one of the strongest echocardiographic predictors of adverse outcomes and warrants comprehensive multiparametric assessment whenever feasible—comprising TAPSE, S’, FAC, RV/LV ratio, and where feasible RV free wall strain. Diastolic dysfunction Grade ≥ 2, frequently coexisting with preserved LVEF, independently predicts mortality and may be evaluated using E/e’ and lateral e’ measurements, with awareness of tachycardia- and ventilator-related limitations.

Therapeutically, early infection control and individualized hemodynamic resuscitation remain the only robustly evidence-based interventions; pharmacological adjuncts, such as beta-blockers and levosimendan, lack convincing clinical trial support. Future research should prioritize standardized SC definitions, phenotype-stratified clinical trials, and multicenter validation of GLS, RV free wall strain, three-dimensional echocardiography, and AI-assisted strain platforms as routine outcome-modifying diagnostic tools.

## Figures and Tables

**Table 1:** Clinical and Echocardiographic Phenotypes of Septic Cardiomyopathy.

Feature	Classic SC (Global Hypokinesia)	Hyperdynamic State	Takotsubo-Like	Diastolic Dysfunction	RV Dysfunction
Echo pattern	Global hypokinesia, LV dilatation	EF > 70%, small LV, high CO	Apical ballooning, basal hyperkinesia	E/e’ > 14; lateral e’ < 9 cm/s; LAVI ↑	TAPSE < 17 mm, FAC < 35%, RV/LV ≥ 1, D-sign
Pathomechanism	Cytokines, mitochondrial failure, Ca^2+^ dysregulation	Vasoplegic afterload reduction, β-adrenergic desensitization	Catecholamine excess (β2-apical dominance)	SERCA2a impairment, increased LV stiffness, inflammatory mediators	Hypoxic vasoconstriction, ARDS, ventilator-induced afterload, direct depression
Reversibility	7–10 days	With vasopressor therapy	Days to weeks	Often persists into early recovery; tracks with infection control	Resolves with PVR reduction and infection control
Short-term prognosis	Neutral (Frank–Starling compensation)	Unfavorable (U-shaped mortality curve)	Favorable (0–8% in-hospital mortality)	Elevated; independent mortality predictor	Markedly elevated; strongest independent predictor
Key differentiator	Exclude ACS; no regional WMA	Exclude hypovolemia (IVC assessment)	Negative coronary angiogram required	Distinguish acute from pre-existing HFpEF	Distinguish acute septic RV failure from PE/chronic cor pulmonale

SC = septic cardiomyopathy; EF = ejection fraction; LV = left ventricle; RV = right ventricle; CO = cardiac output; ACS = acute coronary syndrome; WMA = wall motion abnormality; IVC = inferior vena cava; LAVI = left atrial volume index; SERCA2a = sarcoplasmic Ca^2+^-ATPase; HFpEF = heart failure with preserved ejection fraction; PE = pulmonary embolism; PVR = pulmonary vascular resistance; TAPSE = tricuspid annular plane systolic excursion; FAC = fractional area change.

**Table 2:** Prognostic Profile of Echocardiographic Phenotypes in Septic Cardiomyopathy.

Phenotype	Frequency	Mortality Association	Underlying Mechanism
Global hypokinesia (EF ≈ 45%)	30–40%	Neutral short-term	Compensatory LV dilatation; Frank–Starling adaptation
Hyperdynamic (EF ≈ 75%)	15–20%	Elevated (U-shaped curve)	Marker of severe vasoplegic shock; not preserved cardiac reserve
RV dysfunction	20–50%	Elevated; strong independent predictor	Ventricular interdependence; interventricular septal shift; RV ischemia
Diastolic dysfunction Grade ≥ 2	40–60%	Elevated; independent predictor	Elevated filling pressures; reduced myocardial reserve
Biventricular dysfunction	10–20%	Markedly elevated	Severe multi-organ involvement; biventricular failure cascade

EF = ejection fraction; LV = left ventricle; RV = right ventricle.

**Table 3:** Pathophysiological Mechanisms of Septic Cardiomyopathy.

Mechanism	Molecular Pathway	Clinical Consequence
PAMPs/DAMPs → TLR activation	Cytokine storm → iNOS → NO/peroxynitrite	Direct myocardial suppression; negative inotropy
Mitochondrial dysfunction	Complex I–IV inhibition by ROS; ATP depletion	Contractile failure (reversible in survivors via mitophagy)
Ca^2+^ dysregulation	SERCA2a ↓; L-type Ca^2+^ channel reduction; ↓ troponin sensitivity	Impaired excitation–contraction coupling; prolonged relaxation
Beta-adrenergic desensitization	β1/β2 receptor downregulation; adenylate cyclase uncoupling	Catecholamine resistance; hyperdynamic physiology
Microvascular dysfunction	Endothelial injury; microthrombi; impaired vasoreactivity	Subendocardial ischemia despite elevated macro-coronary flow
Frank–Starling adaptation	LV dilatation → preload augmentation → maintained SV	Short-term cardioprotective; limits excess acute mortality

iNOS = inducible nitric oxide synthase; SERCA2a = sarcoplasmic Ca^2+^-ATPase; SV = stroke volume; LV = left ventricle; ROS = reactive oxygen species; PAMPs = pathogen-associated molecular patterns; DAMPs = damage-associated molecular patterns; TLR = Toll-like receptor.

**Table 4:** Expert opinion-based interpretive framework for GLS values in the ICU setting.

GLS Range	Interpretation	Suggested Considerations
−18% to −22%	Normal longitudinal LV function	Routine clinical monitoring; reassessment at 24–48 h.
−15% to −17%	Subclinical dysfunction; heightened vigilance warranted	Close serial monitoring; biomarker trending; consider full multiparametric echocardiography.
−12% to −14%	Manifest longitudinal dysfunction	Re-evaluate loading conditions and broader echocardiographic phenotype before therapeutic adjustment; targeted intervention based on GLS alone is not currently supported by interventional evidence.
>−12%	Severe longitudinal dysfunction; elevated mortality risk in observational studies	Comprehensive monitoring; phenotype-guided clinical decision-making in the context of full hemodynamic assessment.

GLS = global longitudinal strain; LV = left ventricle. Fewer negative values indicate worse function. The ranges and suggested considerations in this table represent an expert opinion-based interpretive framework derived from observational and prognostic studies; no GLS-guided interventional trial has been conducted in sepsis. GLS values are subject to vendor-specific algorithmic variability; thresholds should be applied with caution when measurements are obtained on a different platform from that used in the underlying validation studies. See EACVI/ASE/Industry Task Force consensus and the EACVI/ASE Inter-Vendor Comparison Study for technical standards [42,43].

**Table 5:** Echocardiographic Parameters for RV Function Assessment in Sepsis.

Parameter	Normal Value	Pathological Threshold	Method/Key Note
TAPSE	≥17 mm	<17 mm	M-mode; preload-dependent; falsely normal with RV volume overload
S’ (TDI)	≥9.5 cm/s	<9.5 cm/s	Lateral tricuspid annulus; less load-dependent; angle-dependent
FAC	≥35%	<35%	Apical 4-chamber area measurement; captures radial component
RV/LV ratio	<2/3	≥1 (RV ≥ LV)	Focused RV-centered A4C; beware apex-tilted transducer artifacts
D-sign (PSAX)	Absent	Present	Systolic = pressure overload; diastolic = volume overload
RVSP (from TRV)	<35 mmHg	>35 mmHg	Peak TR velocity + estimated RAP; 60-60 rule for acute PE
PA acceleration time	>100 ms	<60 ms (notch)	94% specificity for acute PE combined with PASP 30–60 mmHg
RV Free Wall Strain	−20% to −28%	>−20%	STE; sensitive for subclinical RV dysfunction; feasibility in ICU 60–80% (see Section 6.5)

TAPSE = tricuspid annular plane systolic excursion; TDI = tissue Doppler imaging; FAC = fractional area change; PSAX = parasternal short-axis; RVSP = RV systolic pressure; TR = tricuspid regurgitation; RAP = right atrial pressure; PA = pulmonary artery; STE = speckle-tracking echocardiography; A4C = apical four-chamber view; PE = pulmonary embolism; PASP = pulmonary artery systolic pressure.

**Table 6:** Structured Echocardiographic Protocol for Septic Cardiomyopathy Assessment.

Parameter	Normal Range	Pathological in SC	Method	ICU-Specific Feasibility
LVEF (biplane Simpson)	≥55%	<50%, acutely reduced	2D or 3D echocardiography	High; biplane often limited by acoustic window
GLS (Speckle-Tracking)	−18% to −22%	>−18% (less negative)	STE, three apical planes	Moderate; vendor variability; feasibility 70–85%
LVOT-VTI/Stroke Volume	18–22 cm	<18 cm (reduced SV)	Pulsed-wave Doppler at LVOT	High; reproducible
E/e’ ratio	<8 (lateral)	>14 (elevated filling pressures)	TDI annular velocity + PW mitral inflow	Limited at HR > 90–100/min (E–A fusion)
Lateral e’	>9 cm/s	<9 cm/s	TDI at lateral mitral annulus	Generally feasible
TAPSE	≥17 mm	<17 mm	M-mode at lateral tricuspid annulus	High; quick to acquire
S’ (RV-TDI)	≥9.5 cm/s	<9.5 cm/s	TDI at lateral tricuspid annulus	Generally feasible
FAC (RV)	≥35%	<35%	Apical 4-chamber; planimetric	Moderate; depends on endocardial border definition
RV Free Wall Strain	−20% to −28%	>−20%	Speckle-tracking; RV-focused A4C	Lower; feasibility 60–80%; expertise required
IVC diameter/collapse	<21 mm; >50% collapse	Rigid > 21 mm; <50% collapse	M-mode subcostal	Reduced reliability under mechanical ventilation

LVEF = left ventricular ejection fraction; GLS = global longitudinal strain; STE = speckle-tracking echocardiography; LVOT = left ventricular outflow tract; VTI = velocity-time integral; SV = stroke volume; TDI = tissue Doppler imaging; PW = pulsed-wave; HR = heart rate; TAPSE = tricuspid annular plane systolic excursion; FAC = fractional area change; RV = right ventricle; A4C = apical four-chamber view; IVC = inferior vena cava.

## Data Availability

No new data were created or analyzed in this study.

## Supplementary Materials

The following supporting information can be downloaded at: https://www.mdpi.com/article/10.3390/diagnostics16111664/s1, Supplementary Table S1: Key Included Clinical Studies; Supplementary File S1: PRISMA-ScR Flow Diagram and Checklist; Supplementary Table S2: Evidence Grading of the Therapeutic Branches in Figure 1. Ref. [70] has been cited in Supplementary Materials.
