# Supplementary material for: Septic Cardiomyopathy in the ICU: Echocardiographic Phenotypes, Global Longitudinal Strain, and Right Ventricular Assessment
Source: Diagnostics (Basel). 2026 May 28;16(11):1664. doi: 10.3390/diagnostics16111664 (PMC13256233; doi:10.3390/diagnostics16111664)
Supplement: Supplementary file 1 [file diagnostics-16-01664-s001.zip › diagnostics-4303426-supplementary.pdf]

Supplementary Table S1

Key Included Clinical Studies — Septic Cardiomyopathy

"Septic Cardiomyopathy in the ICU: Echocardiographic Phenotypes, Global Longitudinal Strain, and Right Ventricular Assessment"

This table summarises the principal clinical studies, meta-analyses, and translational reviews that constitute the core empirical evidence base of the present narrative review. Included are studies that contributed substantively to the description of phenotypes, prognosis, pathophysiology, or echocardiographic methodology of septic cardiomyopathy. Reference numbers in square brackets refer to the renumbered reference list in the revised manuscript.

Study (first author)	Year	Study design / population	Sample size	Key findings relevant to the review	Role in this review (manuscript section / reference)
Parker et al.	1984	Prospective observational, septic shock	20	First description of profound but reversible biventricular dysfunction in septic shock; LV dilatation in survivors with low EF.	Historical foundational reference for septic cardiomyopathy [4].
Poelaert et al.	1997	Prospective observational, septic shock	30	Coexistence of systolic and diastolic dysfunction in septic shock; diastolic abnormalities frequent independent of EF.	Early evidence of diastolic dysfunction in sepsis [15].
Pulido et al.	2012	Prospective cohort, severe sepsis / septic shock	106	Echocardiographic phenotype unstable: changed in 89% of hypokinetic and 86% of hyperdynamic patients between admission and discharge.	Phenotypic instability — Section 4.2 [24].
Huang et al.	2013	Meta-analysis	ca. 800	Early ventricular dysfunction or LV dilatation associated with lower mortality (Frank–Starling adaptation hypothesis).	Frank–Starling concept; Section 4.2, 5.4 [19].
Sevilla Berrios et al.	2014	Retrospective cohort, severe sepsis / septic shock	274	Low LVEF not associated with 30-day mortality.	Supports neutral mortality of isolated LV dysfunction [21].

Study (first author)	Year	Study design / population	Sample size	Key findings relevant to the review	Role in this review (manuscript section / reference)
Hestenes et al.	2014	Experimental (porcine sepsis)	—	Strain abnormalities precede LVEF decline by 12–24 h in serial assessment.	Temporal advantage of GLS over LVEF [45].
Landesberg et al.	2014	Prospective cohort, severe sepsis / septic shock	262	Troponin elevation in 85–95% of sepsis patients; associated with diastolic dysfunction and RV dilatation; prognostic.	Biomarker section, Section 8 [60].
Sanfilippo et al.	2015	Systematic review and meta-analysis	—	Diastolic dysfunction Grade ≥2 independent predictor of mortality across multiple cohorts.	Diastolic dysfunction prognosis; Section 4.2, 6.4 [14].
Chang et al.	2015	Prospective cohort, septic shock	93	GLS independently associated with mortality in septic shock, beyond LVEF.	GLS prognostic role; Section 6.2 [47].
Ng et al. (SPECKSS)	2016	Prospective case-control, septic shock	60	GLS impaired in septic shock despite preserved LVEF; subclinical longitudinal dysfunction.	Hidden strain loss; Section 6.2 [44].
Boissier et al.	2017	Prospective cohort, septic shock	100	LV dysfunction in septic shock strongly load-dependent; LVEF improves with afterload reduction.	Load dependency of LVEF; Section 6.1, 6.2 [34].
Lanspa et al. (Utah)	2021	Prospective cohort, early sepsis / septic shock	393	RV dysfunction associated with 3.4-fold increase in 28-day mortality; LV dysfunction not independently associated.	Cornerstone of RV prognostic argument; Section 4.2, 6.3.2 [12].
Vallabhajosyula et al.	2021	Meta-analysis	1,373 (10 studies)	RV dysfunction associated with 2.4-fold short-term and 2.3-fold long-term mortality.	Quantitative summary of RV impact; Section 4.2, 6.3.2 [13].

Study (first author)	Year	Study design / population	Sample size	Key findings relevant to the review	Role in this review (manuscript section / reference)
Dugar et al.	2023	Retrospective cohort	385	Isolated LV systolic dysfunction not independently associated with mortality in sepsis.	Confirms neutral short-term mortality of hypokinetic SC; Section 4.2 [20].
Hendrickson et al.	2024	Retrospective cohort	—	Predictors and mortality rates of septic cardiomyopathy and sepsis-related cardiogenic shock.	Phenotype-specific outcomes; Section 4.2 [23].
Wang et al.	2025	Systematic review and meta-analysis	—	Time course of morbidity and mortality across phenotypes in sepsis; underestimation of incidence in early echo studies.	Timing of echocardiographic assessment; Section 4.1 [22].
Sato et al. (esmolol/landiolol)	2025	Systematic review with TSA	—	No robust mortality benefit from beta-blockade in sepsis; risk of hemodynamic deterioration.	Section 9.3 [66].
Huespe et al.	2026	Retrospective multicentre cohort	—	Abnormal GLS at ICU admission independently predicts long-term outcomes in sepsis.	GLS long-term prognosis; Section 6.2 [46].
Frapard et al.	2026	Translational systematic review and meta-analysis	—	Cytokine-mediated mechanisms of septic myocardial dysfunction; TNF-α, IL-1β, IL-6, NO.	Pathophysiology, Section 5.1 [28].

Abbreviations: EF = ejection fraction; GLS = global longitudinal strain; ICU = intensive care unit; LV = left ventricle; LVEF = left ventricular ejection fraction; NO = nitric oxide; RV = right ventricle; SC = septic cardiomyopathy; TSA = trial sequential analysis.

Note: *This table presents a structured selection of pivotal studies and is not an exhaustive enumeration of all references cited in the manuscript. Foundational guideline documents (e.g., Surviving Sepsis Campaign 2021, Sepsis-3, ASE/EACVI chamber quantification and diastolic function recommendations) and mechanistic/pathophysiological reviews are cited in the main reference list.*

Supplementary File S1

PRISMA-ScR Flow Diagram and Checklist

Part 1 — Flow Diagram of Literature Identification, Screening and Inclusion

Figure S1-1 below summarises the structured workflow used to identify, screen, and include literature for this narrative review. Although a fully systematic review approach was not applied (this is a narrative review, not a scoping or systematic review), the workflow follows the principles of PRISMA-ScR for transparency.

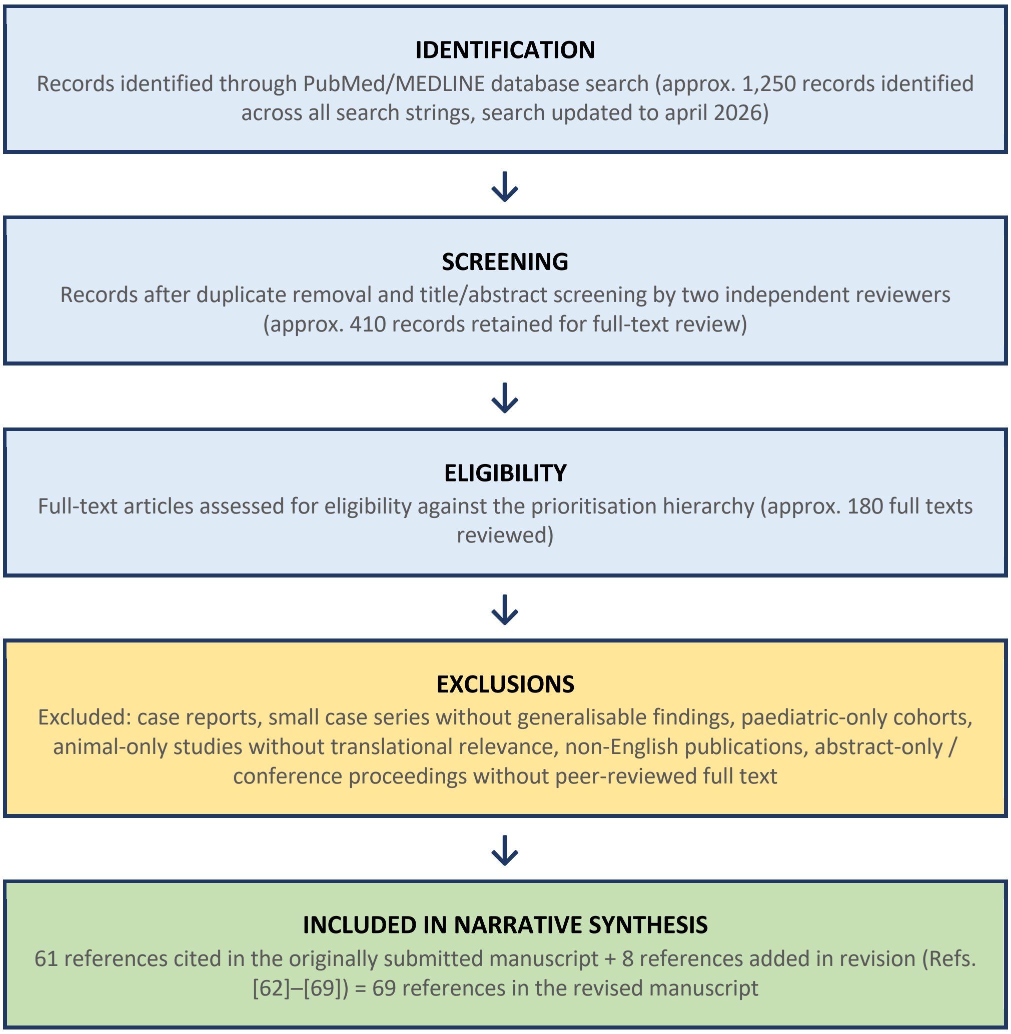

Figure S1-1. *Flow diagram of the literature search and selection process used in this narrative review. The number of records at each stage represents an approximate count over the cumulative searches across the seven structured search strings listed in the Methods section of the main manuscript.*

Part 2 — PRISMA-ScR Checklist (Tricco et al., 2018)

The following checklist documents how each PRISMA-ScR item is addressed in the present narrative review. The checklist follows the PRISMA-ScR statement (Tricco AC et al. PRISMA Extension for Scoping Reviews [PRISMA-ScR]: Checklist and Explanation. Ann Intern Med. 2018;169(7):467–473). Where an item is not formally applicable to a narrative review (e.g., risk of bias assessment of individual sources of evidence, formal quantitative synthesis, registration), this is explicitly stated.

Section	Item	Description / Reporting in this manuscript	Reported on (page/section)
TITLE	1. Title	Identifies the report as a narrative review.	Title page

Section	Item	Description / Reporting in this manuscript	Reported on (page/section)
ABSTRACT	2. Structured summary	Structured abstract (Background/Objectives, Methods, Results, Conclusions, Keywords).	Abstract
INTRODUCTION	3. Rationale	Describes the rationale for the review in the context of septic cardiomyopathy and contemporary echocardiographic phenotyping.	Introduction (Section 1)
	4. Objectives	States the review objectives (synthesis of pathophysiology, phenotypes, GLS, RV assessment, diastolic dysfunction, and therapeutic implications).	End of Section 1
METHODS	5. Protocol and registration	Not applicable. As a narrative review, no a priori protocol was registered.	—
	6. Eligibility criteria	Eligible study types: original research articles (prospective and retrospective), systematic reviews and meta-analyses, clinical practice guidelines and consensus statements, and translational studies of high clinical relevance. Exclusions: case reports, non-generalisable case series, paediatric-only cohorts (where adult-relevant data unavailable), purely animal studies without translational relevance, non-English publications. Time frame: predominantly 2010–March 2026; seminal historical references included for mechanistic/historical context.	Section 2 (Methods)
	7. Information sources	PubMed/MEDLINE; date of last search update: March 2026.	Section 2 (Methods)
	8. Search	Boolean search strings: "septic cardiomyopathy," "sepsis-induced myocardial dysfunction," "global longitudinal strain sepsis," "right ventricular dysfunction sepsis," "diastolic dysfunction sepsis," "speckle-tracking echocardiography sepsis," and "hemodynamic monitoring sepsis."	Section 2 (Methods)
	9. Selection of sources of evidence	Two-stage screening: (i) independent title/abstract screening by two authors; (ii) full-text review of potentially eligible records. Disagreements resolved by consensus discussion among all authors.	Section 2 (Methods)
	10. Data charting process	Qualitative narrative synthesis. Key clinical studies summarised in Supplementary Table S1.	Section 2 (Methods); Suppl. Table S1
	11. Data items	For each included study: design, population, sample size, principal echocardiographic and outcome variables.	Suppl. Table S1
	12. Critical appraisal of individual sources of evidence	Not formally applicable for a narrative review. Methodological priority hierarchy explicitly stated (high-quality systematic reviews/meta-analyses → RCTs and large multicentre cohorts → smaller prospective cohorts → mechanistic studies of high relevance).	Section 2 (Methods)
	13. Synthesis of results	Qualitative narrative synthesis. No quantitative meta-analytic synthesis was performed given heterogeneity of populations, definitions, and outcomes.	Section 2; Section 10.3
RESULTS	14. Selection of sources of evidence	Approximately 1,250 records identified, ~410 retained after title/abstract screening, ~180 full-text reviewed, 69 included in the synthesis.	Suppl. Figure S2-1
	15. Characteristics of sources of evidence	Summarised in Supplementary Table S1.	Suppl. Table S1

Section	Item	Description / Reporting in this manuscript	Reported on (page/section)
	16. Critical appraisal	Not formally applied. See item 12.	—
	17. Results of individual sources of evidence	Reported throughout the body of the narrative review with relevant references.	Sections 3–9
	18. Synthesis of results	Narrative synthesis structured by topic (definitions, epidemiology, pathophysiology, echocardiographic assessment, diagnostic algorithm, biomarkers, therapy).	Sections 3–9
DISCUSSION	19. Summary of evidence	Provided in Section 10.1 (Interpretation of Key Findings) and Section 10.2 (Clinical Implications).	Sections 10.1, 10.2
	20. Limitations	Limitations of narrative design, absence of meta-analytic synthesis, lack of universally accepted SC definition, vendor variability of GLS, and limited phenotype-guided interventional evidence are explicitly addressed.	Section 10.3
	21. Conclusions	Provided in Section 11.	Section 11
FUNDING	22. Funding	No external funding was received for the preparation of this review.	Funding statement

Reference: *Tricco AC, Lillie E, Zarin W, et al. PRISMA Extension for Scoping Reviews (PRISMA-ScR): Checklist and Explanation. Ann Intern Med. 2018;169(7):467–473.*

Supplementary Table S2

Evidence Grading of the Therapeutic Branches in Figure 1

This table assigns an explicit evidence category to each therapeutic node of the phenotype-guided algorithm presented in Figure 1 of the main manuscript. Three categories are used: **Evidence-supported** (recommendation backed by guideline-grade evidence or robust randomised controlled trials/meta-analyses), **Physiology-based / expert opinion** (recommendation derived from pathophysiological reasoning, observational data, or expert consensus, but not yet supported by phenotype-stratified outcome trials), and **Negative / insufficient trial evidence** (interventions for which dedicated trials have failed to demonstrate benefit or have shown signals of harm). The colour coding of the table rows mirrors this categorisation.

Colour legend: ■ Evidence-supported ■ Physiology-based / expert opinion ■ Negative / insufficient trial evidence

Phenotype (Figure 1)	Therapeutic node	Evidence category	Justification / supporting evidence	Reference(s) in revised manuscript
All phenotypes	Early infection control: broad-spectrum antimicrobial therapy ≤ 1 h + source control	**Evidence-supported**	Cornerstone of sepsis management per Surviving Sepsis Campaign 2021 guidelines (strong recommendation, moderate-to-high quality evidence). Myocardial recovery closely parallels infection resolution.	[62]
Hypokinetic (LVEF↓ / GLS↓ / LV dilatation)	Norepinephrine first-line, MAP target ≥ 65 mmHg	**Evidence-supported**	First-line vasopressor in septic shock per Surviving Sepsis Campaign 2021 (strong recommendation, high-quality evidence).	[62]
Hypokinetic	Dobutamine for inotropic support if reduced cardiac output is confirmed	**Physiology-based / expert opinion**	Conditional Surviving Sepsis Campaign 2021 recommendation in case of persistent hypoperfusion despite adequate fluid status and MAP. No phenotype-stratified RCT in sepsis. Risk of tachycardia, increased myocardial oxygen demand and arrhythmia documented; contraindicated in hyperdynamic SC.	[17,18,62]
Hyperdynamic (EF↑ / SVR↓ / vasoplegic shock)	Norepinephrine for SVR restoration; MAP ≥ 65 mmHg	**Evidence-supported**	Same as for hypokinetic phenotype. Particularly critical in hyperdynamic SC where supranormal LVEF reflects severe vasoplegic shock rather than preserved myocardial reserve.	[62]
Hyperdynamic	Add vasopressin for norepinephrine-sparing effect	**Evidence-supported**	Conditional Surviving Sepsis Campaign 2021 recommendation as adjunctive vasopressor; reduces norepinephrine requirements without convincing mortality benefit.	[62]
Hyperdynamic	Avoid inotropic support (dobutamine contraindicated)	**Physiology-based / expert opinion**	Pathophysiologically grounded: in hyperdynamic SC, supranormal LVEF reflects vasoplegia, not preserved	[17,18]

Phenotype (Figure 1)	Therapeutic node	Evidence category	Justification / supporting evidence	Reference(s) in revised manuscript
			cardiac reserve; inotropy may worsen tachycardia and pulmonary perfusion mismatch.	
RV failure (TAPSE↓ / FAC↓ / RV/LV ratio ≥ 1)	RV-protective ventilation: minimise PEEP and driving pressure	**Physiology-based / expert opinion**	Strong pathophysiological and observational evidence in ARDS, extrapolated to septic RV failure. Repessé et al. and Vieillard-Baron expert consensus.	[54,68]
RV failure	Inhaled NO or prostacyclin for elevated pulmonary vascular resistance	**Physiology-based / expert opinion**	Reduces RV afterload acutely; effect on outcome not demonstrated in dedicated sepsis trials. Use guided by clinical context and bedside echocardiographic response.	[52,55]
RV failure	Milrinone (alternatively dobutamine) for impaired RV contractility	**Physiology-based / expert opinion**	Pulmonary vasodilator and inotropic effects favourable in RV dysfunction; risk of systemic hypotension. Outcome impact in septic RV failure not established.	[55]
RV failure	Veno-arterial ECMO as bridge-to-recovery in refractory cases	**Physiology-based / expert opinion**	May be considered when source control achieved and recovery anticipated. Evidence base for sepsis-related cardiogenic shock remains limited; selection criteria and timing under active investigation.	[57]
Diastolic dysfunction (E/e′ > 14 / Grade ≥ 2)	Restrictive volume strategy; E/e′ monitoring after fluid challenges	**Physiology-based / expert opinion**	Driven by elevated filling pressures and risk of pulmonary oedema. Supported by observational data; no randomised phenotype-stratified trial of E/e′-guided fluid strategy in sepsis.	[14,58]
All phenotypes	Dynamic fluid responsiveness testing (PLR, EEO, ΔVTI) in preference to static measures	**Evidence-supported**	Robust meta-analytic evidence of superior diagnostic performance vs. static measures (CVP, IVC alone). Monnet et al. meta-analysis (PLR-cCO pooled AUC ~0.95).	[36,63]
All phenotypes (vasopressor-dependent shock)	Hydrocortisone in vasopressor-dependent septic shock	**Evidence-supported**	Reduces vasopressor duration and requirements; updated 2024 corticosteroid guideline and Cochrane 2025 meta-analysis support short-course hydrocortisone in vasopressor-dependent septic shock.	[64,65]
All phenotypes (sepsis-related tachycardia)	Beta-blockers (esmolol, landiolol)	**Negative / insufficient trial evidence**	Initial single-centre RCT signal (Morelli 2013, esmolol) not confirmed in subsequent multicentre trials including STRESS-L. Recent Sato 2025 systematic review with trial sequential analysis: no robust mortality benefit; signal of haemodynamic deterioration. Not currently recommended outside clinical trials.	[66]

Phenotype (Figure 1)	Therapeutic node	Evidence category	Justification / supporting evidence	Reference(s) in revised manuscript
All phenotypes (LV systolic dysfunction)	Levosimendan	**Negative / insufficient trial evidence**	LeoPARDS RCT and subgroup analysis: no mortality benefit in septic shock. Not currently recommended as standard therapy for septic cardiomyopathy.	[67]

Abbreviations: ECMO = extracorporeal membrane oxygenation; EEO = end-expiratory occlusion; FAC = fractional area change; GLS = global longitudinal strain; LV = left ventricle; LVEF = left ventricular ejection fraction; MAP = mean arterial pressure; NO = nitric oxide; PEEP = positive end-expiratory pressure; PLR = passive leg raise; RCT = randomised controlled trial; RV = right ventricle; SC = septic cardiomyopathy; SVR = systemic vascular resistance; TAPSE = tricuspid annular plane systolic excursion; VTI = velocity-time integral.

Note: *The algorithm in Figure 1 represents a pragmatic, expert-based conceptual framework that has not been prospectively validated in interventional trials. The evidence categories assigned in this table reflect the editorial judgement of the authors based on the cited evidence and should be interpreted in conjunction with the limitations discussed in Section 10.3 of the main manuscript.*
